# An Inverse Signorini Obstacle Problem

**DOI:** 10.1007/s00205-026-02197-1

**Published:** 2026-05-26

**Authors:** Maarten V. de Hoop, Matti Lassas, Jinpeng Lu, Lauri Oksanen, Ziyao Zhao

**Affiliations:** 1 Computational and Applied Mathematics and Earth Science, Rice University, Houston, TX 77005 USA; 2 Department of Mathematics and Statistics, University of Helsinki, Helsinki, 00014 Finland

## Abstract

We study the inverse problem of determining a Signorini obstacle from boundary measurements for the isotropic elasticity system. We prove that the obstacle can be uniquely determined by a single measurement of displacement and normal stress for the Signorini problem on an open subset of the boundary up to a natural obstruction. In addition to considering the Signorini problem, we develop techniques that can be used to study inverse problems for general differential inequalities.

## Introduction

The Signorini problem is a classical free boundary problem originally introduced by A. Signorini in the field of linear elasticity [47]. The problem is characterized by the boundary conditions that the solution of the problem at each boundary point must satisfy one of two possible boundary conditions, without a prior knowledge of which condition applies to each point. Physically, the Signorini conditions model frictionless contact between an elastic body and a rigid support, and they are formulated in terms of inequalities and a nonlinear complementarity condition. While the original Signorini problem was formulated for the elasticity system, its scalar version has been widely studied as a classical variational problem arising from applications [18, 32]. In this paper, we study the inverse problem of determining a Signorini obstacle from a single boundary measurement for the isotropic elasticity system. We start by formulating the scalar version of our problem, and then turn to the problem in linear elasticity.

### Scalar Version

Let $\Omega \subset \mathbb {R}^n$ be a bounded connected open set with smooth boundary, and $O\subset \subset \Omega$ be a connected open subset with smooth boundary modelling an obstacle. The scalar Signorini problem for the Laplace equation on $\Omega \setminus {\overline{O}}$ is formulated as follows, with smooth boundary data *f* on the exterior boundary $\partial \Omega$:1Here, the normal derivative $\partial _{\nu } u|_{\partial O}$ is taken with respect to the outward unit normal $\boldsymbol{\nu }$ for $\Omega \setminus {\overline{O}}$ at $\partial O$. This is also known as the thin obstacle problem that appears in studying classical obstacle problems [22, 24, 45] when the constraint is only imposed for the boundary (a hypersurface).

In general the Signorini problem (1) does not have smooth solutions up to the boundary of the obstacle. The weak formulation of the problem (1) is understood as a variational problem of finding $u\in K$ where

$$ K:=\big \{v\in H^1(U): v|_{\partial O} \ge 0, \; v|_{\partial \Omega }=f \big \},\quad U:=\Omega \setminus {\overline{O}}, $$

such that

$$\begin{aligned} \int _{U} \nabla u \cdot \nabla (v-u) \textrm{d}x \ge 0, \; \, \text { for all }v\in K. \end{aligned}$$

A general theory for the existence and uniqueness of solution to the variational problem was developed by Lions-Stampacchia in [36]. It was known due to Frehse [25] and Caffarelli [9] that the solution to the scalar Signorini problem is in $H^2(U)\cap C^{1,\alpha }({\overline{U}})$ for some $\alpha \le 1/2$. The optimal $C^{1,\frac{1}{2}}$-regularity was proved years later by Athanasopoulos-Caffarelli [5].

We consider an inverse obstacle problem: given a boundary value *f*, does the normal derivative $\partial _{n} u|_{\Gamma }$ on an open subset $\Gamma \subset \partial \Omega$ of the exterior boundary $\partial \Omega$ uniquely determine the obstacle *O*? Observe that the inverse problem cannot be uniquely solved when the given boundary value *f* is a nonnegative constant function, in which case *u* is the same (nonnegative) constant everywhere so the normal derivative is identically zero no matter what the obstacle is. We prove that this is the only obstruction in solving the inverse obstacle problem.
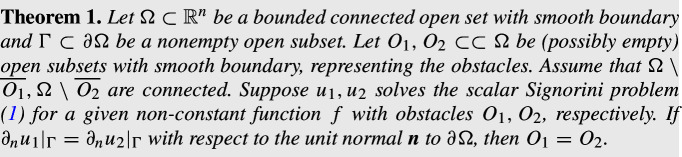


#### Remark 1.1

As a special case of Theorem 1, the boundary data determine if there is an (smooth) obstacle or not. The conclusion of Theorem 1 is still valid if *f* is a negative constant, which is stated as Proposition 6.1.

### Linear Elasticity

Consider that an elastic body occupying $\Omega \setminus {\overline{O}}$, in an equilibrium configuration, is constrained on $\partial \Omega$ and rests on a frictionless rigid body *O*. Let us denote by ${\boldsymbol{u}}:\Omega \setminus {\overline{O}}\rightarrow \mathbb {R}^n$ the displacement vector of the elastic body and by $\boldsymbol{\varepsilon }({\boldsymbol{u}})=\frac{1}{2}(\nabla {\boldsymbol{u}}+(\nabla {\boldsymbol{u}})^T)$ the linearized strain tensor. Then the stress tensor for the Lamé system is given by

$$\begin{aligned} \boldsymbol{\sigma }({\boldsymbol{u}})=2\mu \boldsymbol{\varepsilon }({\boldsymbol{u}})+\lambda \, \text {tr}(\boldsymbol{\varepsilon }({\boldsymbol{u}}))I_n, \end{aligned}$$

where $\mu (x),\lambda (x)\in C^\infty ({\overline{\Omega }})$ are positive smooth Lamé coefficients. Let us denote by $\boldsymbol{\nu }$ and ${\boldsymbol{n}}$ the outward unit normal for $\Omega \setminus {\overline{O}}$ at $\partial O$ and $\partial \Omega$, respectively. At each point of $\partial O$, one of the following two conditions holds (see e.g. [18, 48]):

$$\begin{aligned} {\left\{ \begin{array}{ll} \boldsymbol{\sigma }({\boldsymbol{u}})_\tau =0,\ \boldsymbol{\sigma }({\boldsymbol{u}})_\nu \le 0,\ {\boldsymbol{u}}_\nu = 0,\\ \qquad \qquad \qquad \text {or}\\ \boldsymbol{\sigma }({\boldsymbol{u}})_\tau =0,\ \boldsymbol{\sigma }({\boldsymbol{u}})_\nu =0,\ {\boldsymbol{u}}_\nu < 0, \end{array}\right. }. \end{aligned}$$

Here, ${\boldsymbol{u}}_\nu ={\boldsymbol{u}}\cdot \boldsymbol{\nu }$ is the normal displacement, $\boldsymbol{\sigma }({\boldsymbol{u}})_\nu =\boldsymbol{\sigma }({\boldsymbol{u}})\boldsymbol{\nu }\cdot \boldsymbol{\nu }$ is the normal stress, and $\boldsymbol{\sigma }({\boldsymbol{u}})_\tau =\boldsymbol{\sigma }({\boldsymbol{u}})\boldsymbol{\nu }-\boldsymbol{\sigma }({\boldsymbol{u}})_\nu \boldsymbol{\nu }$ is the tangential stress (being zero due to no friction). The first set of conditions above is satisfied in the region where the elastic body is constrained by $\partial O$, meaning that it is in the contact region and no displacement occurs in the normal direction. The second set of conditions holds in the region where normal displacement occurs, indicating the absence of an active obstacle and, consequently, no normal stress. Note that in our setting we turn the typical formulation inside out: a deformable body $\Omega \setminus {\overline{O}}$ surrounding a rigid obstacle *O*. Then the classical formulation of the model, with prescribed smooth displacement ${\boldsymbol{f}}$ on the exterior boundary $\partial \Omega$, is2The Signorini problem in linear elasticity is to find a solution to the system (2).

Similar to the scalar case, the system (2) does not have smooth solutions in general, and the problem is understood as a variational problem of finding ${\boldsymbol{u}}\in {\boldsymbol{K}}$ where

$$\begin{aligned} {\boldsymbol{K}}:=\big \{{\boldsymbol{v}}\in (H^1(U))^n\mid {\boldsymbol{v}}\cdot \nu \le 0 \text { on }\partial O,\ {\boldsymbol{v}}={\boldsymbol{f}}\text { on }\partial \Omega \big \},\quad U:=\Omega \setminus {\overline{O}}, \end{aligned}$$

such that

$$\begin{aligned} \int _{U} \boldsymbol{\sigma }({\boldsymbol{u}}) : (\boldsymbol{\varepsilon }({\boldsymbol{v}})-\boldsymbol{\varepsilon }({\boldsymbol{u}})) \,\textrm{d}x\ge 0,\ \text {for all }{\boldsymbol{v}}\in {\boldsymbol{K}}, \end{aligned}$$

where the operation  :  is defined as $\boldsymbol{\sigma }:\boldsymbol{\varepsilon }=\sum _{i,j=1}^n \sigma _{ij}\varepsilon _{ij}$, where $\sigma _{ij}$, $\varepsilon _{ij}$ are the components of the tensors $\boldsymbol{\sigma },\,\boldsymbol{\varepsilon }$, respectively. The existence and uniqueness of the weak solution to the variational problem above were studied by Fichera [23], see also [36, 48], and the solution is in $H^2(U)$ due to Kinderlehrer [33]. In [46] Schumann proved the $C^{1,\alpha }$-regularity of the solution in general dimensions $n\ge 2$ for some $\alpha >0$. In dimension 3, the optimal $C^{1,\frac{1}{2}}$-regularity was proved by Andersson [3].

**Fig. 1:**
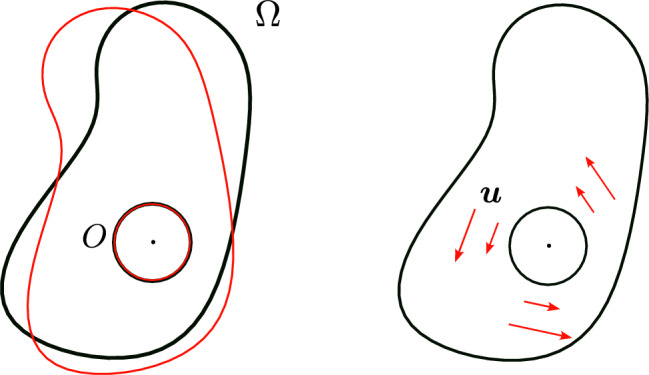
Boundary measurements of the displacement ${\boldsymbol{u}}$ on $\partial \Omega$ resulting from rotations cannot distinguish the size of a round Signorini obstacle *O*, in which case the stress tensor is identically zero. The right figure illustrates the displacement vector ${\boldsymbol{u}}$ resulting from a (infinitesimal) rotation with respect to the center of the obstacle. The displacement on $\partial O$ is perpendicular to the normal direction so the Signorini contact conditions on $\partial O$ are valid.

We study the inverse obstacle problem: given boundary data ${\boldsymbol{f}}$, does the normal stress $\boldsymbol{\sigma }({\boldsymbol{u}}){\boldsymbol{n}}|_{\Gamma }$ on a subset $\Gamma \subset \partial \Omega$ of the exterior boundary $\partial \Omega$ uniquely determine the obstacle *O*? In general this is not possible if the given boundary data ${\boldsymbol{f}}$ is allowed to take the form of a rigid motion defined by

$$\begin{aligned} {\mathcal {R}}:=\big \{{\boldsymbol{c}}+A {\boldsymbol{x}}\mid {\boldsymbol{c}}\in \mathbb {R}^n,\ A\in \mathbb {R}^{n\times n},\ A^T=-A,\ {\boldsymbol{x}}\in \partial \Omega \big \}. \end{aligned}$$

A counterexample to the unique determination is explained in Fig. 1, where the deformation of an elastic body corresponds to a rotation with respect to the center of a round obstacle *O*. The displacement vector is given by ${\boldsymbol{u}}=A{\boldsymbol{x}}$, where *A* is a skew-symmetric matrix and ${\boldsymbol{x}}$ is the position vector. In this case, the Signorini boundary conditions are satisfied on any sphere (with the same center) regardless of its radius, so the radius of the obstacle cannot be detected from measurements on the exterior boundary.

We are able to fully solve the inverse obstacle problem up to this natural obstruction.
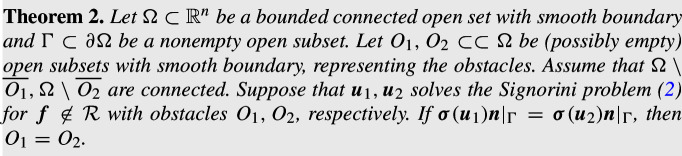


#### Remark 1.2

In the case of ${\boldsymbol{f}}={\boldsymbol{c}}+A{\boldsymbol{x}}\in {\mathcal {R}}$ for some constant vector ${\boldsymbol{c}}\in \mathbb {R}^n$ and skew-symmetric matrix *A*, the solvability of the inverse problem depends on ${\boldsymbol{c}}$ and *A*. For example, when $|{\boldsymbol{c}}|$ is larger than the range of *A*, the inverse obstacle problem is uniquely solvable. A characterization of unique solvability in this case is stated in Proposition 6.2.

We prove Theorem 1 and 2 by arguing that both $O_1\setminus O_2$ and $O_2\setminus O_1$ are empty. We prove this by contradiction, with a unique continuation approach that dates back to M. Schiffer; for details we refer readers to [35, Theorem 5.6] and the remark that follows. The main difficulty lies in the fact that the intersection between two open sets may have rough boundary, even if the two open sets both have smooth boundary. First, the boundary of the intersection is not necessarily piecewise smooth and could have infinitely many connected components. Indeed, this can happen when one of the open set has wildly oscillating boundary of the form $e^{-1/x^2}\sin (1/x)$. Moreover, the set of boundary points at which the unit normal is well-defined can be significantly smaller than the whole boundary, for example considering the disk with a slit: $\{(r,\theta ): 0<r<1,\, 0<\theta <2\pi \}$ in the polar coordinate of $\mathbb {R}^2$. The roughness of the boundary causes essential difficulties in utilizing contact conditions on the difference of the obstacles. To make matters worse, the Signorini contact conditions impose a ceiling on the regularity of the solutions, $C^{1,\alpha }$-regularity to be exact, even if the boundary data are smooth. To handle these difficulties, we operate using the theory of functions of bounded variations and sets of finite perimeter. In Section 3, we briefly review these concepts and show how they apply to our setting. Then we prove our main results in Section 4 and 5.

### Motivations and Related Results

Inverse obstacle problems study the detection of obstacles (or inclusions) from boundary measurements generated by physical fields, such as electrical, acoustic or thermal boundary measurements. There is vast literature on inverse obstacle problems [31] and we focus on theoretical results on these problems for elliptic equations. From single boundary measurement, one of the first uniqueness proofs for identifying obstacles is due to Schiffer [35] originally used in inverse scattering. A more recent work [6] used this method to prove the unique recovery of obstacle with the Robin boundary condition. In the case of many boundary measurements, Isakov proved the unique determination of obstacle and isotropic conductivity from the Dirichlet-to-Neumann map [30]. Logarithmic stability estimate for the determination of obstacle was obtained in [1]. Obstacle detection in isotropic medium using complex geometrical optics solutions was studied in [26, 51, 53]. In the anisotropic case, the unique determination of obstacle was studied in [34] using fundamental solutions for constant medium, and [42] using Runge approximation. Constructive methods to find obstacles include [8] for single measurement and [27] for many measurements.

The Signorini contact condition considered in the present work is a classical way to model frictionless contact of an elastic body. It defines a free boundary problem in the sense that the actual region of contact is not known a priori and needs to be determined as part of the problem. Free boundary problems naturally occur in physics, for instance in fluid dynamics, contact and fracture mechanics [18, 20, 32]. When friction is present, in the dynamic setting, the analysis of contact models becomes more complicated. For (visco)elastic bodies, a physically reasonable Coulomb friction law has been defined in a nonlocal way by mollifying the normal force, which guarantees well-posedness; see [50] for a detailed model including the Signorini contact condition.

In general, these problems can be mathematically understood through variational inequalities (or minimization of energy functionals) over a set of constraints, giving rise to a system of differential inequalities instead of the classical Euler-Lagrange equations. Inverse problems have been extensively studied from the perspective of PDEs and differential geometry, but are almost unknown for differential inequalities mainly due to their nonlinear and complex nature. To the best of our knowledge, our present paper serves as a first study into inverse problems for free boundary contact models. As many physically relevant problems involve contact conditions, the classical inverse problems for PDEs could have generalizations to (partial) differential inequalities. We hope that the present paper initiates new research directions and develops techniques suitable for the future study of inverse problems for (partial) differential inequalities.

More traditional inverse problems for the constitutive parameters of elastic materials have a long history. Inverse problems of determining the stiffness tensor in the elasticity system from boundary measurements have been widely studied for both static and dynamic cases. For the static problem, the determination is only known for isotropic cases when the Lamé parameters are close to constants [21, 29, 39–41], and the stability was analyzed in [7]. For the dynamic problem, the unique determination of stiffness tensor is known for isotropic elasticity [15, 43, 44, 49]. The dynamic inverse rupture problem and a related inverse fault friction problem in an isotropic elastic medium, and their stability, were analyzed in [13, 14]. The determination of anisotropic elasticity was studied in [10, 16, 17, 38] and recent works [12, 28].

## Preliminary Constructions

**Fig. 2:**
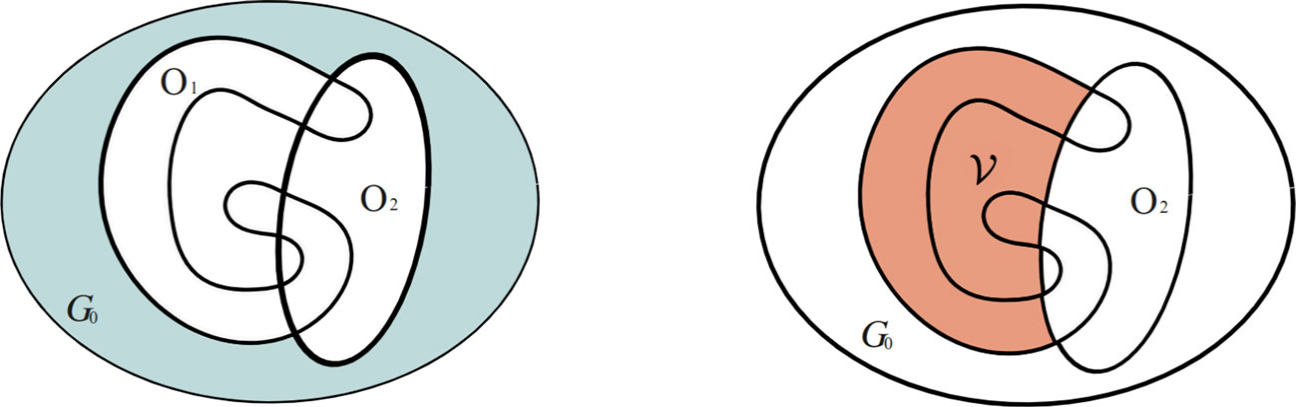
An illustration of the sets $G_0$ and ${\mathcal {V}}$

The basic idea of our method is to consider the elasticity system on the difference of the obstacles $O_1\setminus O_2$ with the Signorini conditions on its boundary, and produce a contradiction with the exterior boundary measurement through unique continuation. However, as explained above, the set $O_1\setminus O_2$ may be complicated even if $O_1,O_2$ have smooth boundary. Moreover, the complement of $O_1\cup O_2$ may be disconnected, see Fig. 2 (left), which creates an inner region not accessible from the exterior boundary $\partial \Omega$. This makes it difficult to determine the shape in the inaccessible region from exterior boundary measurements.

In this section, we construct appropriate domains to carry out our method. Let $\Omega \subset \mathbb {R}^n$ be a bounded open set with a smooth boundaries, and let $O_1,O_2$ be two open subsets with smooth boundary satisfying that

1. $$O_1\subset \subset \Omega $$ and $$O_2\subset \subset \Omega $$,
2. $$O_1\not \subset O_2$$,
3. $\Omega \setminus \overline{O_1}$ and $\Omega \setminus \overline{O_2}$ are connected.

We define the following sets:

3 $$\begin{aligned} G_0:= \text {the connected component of } \Omega \setminus (\overline{O_1\cup O_2}) \text { such that } \partial \Omega \subset \partial G_0, \end{aligned}$$

4 $$\begin{aligned} {\mathcal {V}}:= \text {a connected component of } (\Omega \setminus \overline{G_0})\setminus \overline{O_2} \text { satisfying } \partial {\mathcal {V}}\cap \partial G_0\ne \emptyset . \end{aligned}$$

An illustration of these sets is given in Fig. 2.

Roughly speaking, $G_0$ represents the largest domain in which unique continuation can be propagated from exterior boundary data, and ${\mathcal {V}}$ is a set containing a connected component of $O_1\setminus \overline{O_2}$ that is connected to the exterior boundary. However, it is not clear from the definitions if there exists a connected component satisfying the definition of ${\mathcal {V}}$. In Lemma 2.3, we show that one can always have a nonempty choice of the set ${\mathcal {V}}$.

### Lemma 2.1

$$\quad \partial G_0\cap (\partial O_1\setminus \partial O_2)\ne \emptyset $$.

### Proof

Suppose $\partial G_0\cap (\partial O_1\setminus \partial O_2)= \emptyset$, then $\partial G_0\subset \partial \Omega \cup \partial O_2=\partial (\Omega \setminus \overline{O_2})$. According to Lemma A1 in Appendix A, there holds $G_0=\Omega \setminus \overline{O_2}$. However, by definition we have $G_0\subset \Omega \setminus (\overline{O_1\cup O_2})=(\Omega \setminus \overline{O_2})\setminus (O_1\setminus \overline{O_2})$ and it is sufficient to show that $O_1\setminus \overline{O_2}\ne \emptyset$. Indeed, suppose $O_1\setminus \overline{O_2}= \emptyset$, then $O_1\subset O_2\cup \partial O_2$ and there exists a point $p\in O_1\cap \partial O_2$ as $O_1\not \subset O_2$. Since $\partial O_2$ is smooth, any neighborhood *U* of *p* intersects $\Omega \setminus \overline{O_2}$ and for sufficiently small neighborhood $U_p$ there holds $U_p\subset O_1$. Thus $\emptyset \ne U_p\cap (\Omega \setminus \overline{O_2})\subset O_1\cap (\Omega \setminus \overline{O_2})$, which contradicts with $O_1\subset \overline{O_2}$. $\square$

### Lemma 2.2

For any point $z\in \partial G_0\cap (\partial O_1\setminus \partial O_2)$, there exists a small neighborhood $U_z$ of *z* such that $U_z\setminus \overline{O_1}\subset G_0$.

### Proof

First we show that $z\not \in \overline{O_2}$. If this is not true, we have $z\in O_2$ as $z\not \in \partial O_2$ by setting. However, this contradicts with $z\in \partial G_0\subset \overline{G_0}\subset {\overline{\Omega }}\setminus (O_1\cup O_2)$.

Since $O_1$ is smooth and $z\in \partial O_1$, for small enough neighborhood $U_z$ of *z* there holds $U_z$ is a local coordinate chart near *z* and $U_z\cap \overline{O_2}=\emptyset$. To simplify the notation, we write $U_z^-:= U_z\setminus \overline{O_1}$. Since $z\in \partial G_0$, there exists a sequence of points $z_j\in G_0$ such that $z_j\rightarrow z$. Then for sufficiently large *J* we have $z_J\in U_z^-$, since $G_0\cap \overline{O_1}=\emptyset$ by definition. Thus, for any $p\in U_z^-$, one can connect *p* with $z_J$ by a path in $U_z^-$.

Since $U_z^-$ does not intersect with $\overline{O_1}$ or $\overline{O_2}$, we see that $p\in G_0$, which proves the statement as $p\in U_z^-$ is arbitrary. $\square$

Lemma 2.1 indicates that $\partial G_0\setminus \partial O_2$ is not empty, which allows us to construct a non-empty ${\mathcal {V}}$.

### Lemma 2.3

There is a nonempty connected component ${\mathcal {V}}$ of $(\Omega \setminus \overline{G_0})\setminus \overline{O_2}$ satisfying $\partial {\mathcal {V}}\cap \partial G_0\ne \emptyset$.

### Proof

According to Lemma 2.1, there exists a point $p\in \partial G_0\cap (\partial O_1\setminus \partial O_2)$, and we can find an open ball $B(p,\delta )$ centered at *p* with radius $\delta$ such that $B(p,\delta )\cap \overline{O_2}=\emptyset$. If such ball does not exist, that is, for any $\varepsilon >0$, there holds $B(p,\varepsilon )\cap \overline{O_2}\ne \emptyset$. This means that $p\in \overline{O_2}$. Notice that $p\not \in \partial O_2$, then $p\in O_2$ and there is an neighborhood $U_p$ of *p* such that $U_p\subset O_2$ and $U_p\cap G_0=\emptyset$, then $p\not \in \partial G_0$, a contradiction.

Furthermore, since $O_1$ is a domain with smooth boundary and $p\in \partial O_1$, there exists $\eta >0$ such that $B(p,\eta )\cap O_1$ is connected by the local connectedness of $\mathbb {R}^n$. Then we choose $\gamma =\min \{\delta ,\eta \}$, thus $\left( B(p,\gamma )\cap (\Omega \setminus \overline{G_0})\right) \subset (\Omega \setminus \overline{G_0})\setminus \overline{O_2}$, and we can let ${\mathcal {V}}$ to be a connected component of $(\Omega \setminus \overline{G_0})\setminus \overline{O_2}$ containing $B(p,\gamma )\cap O_1$. It remains to verify that $p\in \partial {\mathcal {V}}$. Since $p\in \partial O_1$, then we can find a sequence $\{x_n\}_{n\ge 1}\subset O_1$ that converges to *p*, and there exists a number *N* such that $\{x_n\}_{n\ge N}\subset B(p,\gamma )$. Thus $\{x_n\}_{n\ge N}\subset B(p,\gamma )\cap O_1\subset {\mathcal {V}}$, and $p\in {\overline{{\mathcal {V}}}}$. To get a contradiction, assume $p\in {\mathcal {V}}$, then $p\in \Omega \setminus \overline{G_0}$, and in particular $p \not \in \partial G_0$. $\square$

Before concluding this section, we state the following lemma, which plays an important role in proving the main results (the lemma enables us to connect ${\mathcal {V}}$ to $G_0$, as well as $\partial \Omega$, through a path that does not pass $O_2$):

### Lemma 2.4

$$\quad \emptyset \ne \, \partial {\mathcal {V}} \setminus \partial O_2 \subset \partial G_0\cap \partial O_1.$$

### Proof

Notice that

5 $$\begin{aligned} \partial {\mathcal {V}}\subset \partial (\Omega \setminus \overline{G_0})\cup \partial (\Omega \setminus \overline{O_2})\subset \partial \Omega \cup \partial G_0\cup \partial O_2\subset \partial \Omega \cup \partial O_1 \cup \partial O_2. \end{aligned}$$

Since $G_0$ contains a neighborhood of $\partial \Omega$ by the local connectedness, we have $\partial {\mathcal {V}}\cap \partial \Omega =\emptyset$ and $\partial {\mathcal {V}}\subset \partial O_1\cap \partial O_2$. Assume $\partial {\mathcal {V}}\setminus \partial O_2=\emptyset$, then $\partial {\mathcal {V}}\subset \partial O_2\subset \partial (\Omega \setminus \overline{O_2})$. By Lemma A1 in Appendix A, ${\mathcal {V}}=\Omega \setminus \overline{O_2}$ as $\Omega \setminus \overline{O_2}$ is connected, which is a contradiction with $G_0\ne \emptyset$.

Moreover, (5) implies that for any $z\in \partial {\mathcal {V}} \setminus \partial O_2$ satisfies that $z\in \partial (\Omega \setminus G_0)\subset \partial \Omega \cup \partial G_0$. Thus $\partial {\mathcal {V}} \setminus \partial O_2 \subset \partial G_0$ follows immediately from the fact $\partial {\mathcal {V}}\cap \partial \Omega =\emptyset$. $\square$

The boundary of ${\mathcal {V}}$ essentially consists of unions and intersections of manifold boundaries. In general, even when the manifold boundaries are smooth, their unions or intersections can behave wildly, and $\partial {\mathcal {V}}$ is not necessarily piecewise smooth. In fact, ${\mathcal {V}}$ is a set of finite perimeter and the measure-theoretic unit normal on its reduced boundary is well-defined. We will briefly review these concepts in the next section, and show that the measure-theoretic unit normal on the reduced boundary of ${\mathcal {V}}$ coincides with that of manifold boundaries almost everywhere.

## Set of Finite Perimeter and Reduced Boundary

In this section, our primary objective is to show that ${\mathcal {V}}$ is a set of finite perimeter and that its measure-theoretic unit normal coincides with that of $O_1$ or $O_2$, possibly differing by a sign. For self-containedness, we briefly review basic concepts on sets of finite perimeter in geometric measure theory.

Let $u\in L^1(\Omega )$, we say that *u* is a function of bounded variation in $\Omega$, written as $u\in BV(\Omega )$, if its distributional gradient *Du* is a finite $\mathbb {R}^n$ vector-valued Radon measure $Du=(\mu _1,\mu _2,\cdots ,\mu _n)$ such that

$$\begin{aligned} \int _\Omega u\frac{\partial \varphi }{\partial x_i}\textrm{d}x=-\int _\Omega \varphi \textrm{d}\mu _i,\quad \forall \varphi \in C_0^\infty (\Omega ),\ i=1,\cdots ,n, \end{aligned}$$

and

$$\begin{aligned} |Du|(\Omega ):=\sup \left\{ \int _\Omega u\, \text {div }\varphi \ \textrm{d}x\ \big |\ \varphi \in \left( C_0^\infty (\Omega ) \right) ^n,\ \Vert \varphi \Vert _{L^\infty (\Omega )}\le 1 \right\} <\infty . \end{aligned}$$

Let *E* be an measurable subset of $\mathbb {R}^n$. For any open set $\Omega \subset \mathbb {R}^n$, *E* is said to be a set of finite perimeter in $\Omega$ if the characteristic function $\chi _E\in BV(\Omega )$. When *E* is of finite perimeter in $\mathbb {R}^n$, it is simply called a set of finite perimeter.

For a set *E* of finite perimeter, the *reduced boundary* of *E*, denoted by $\partial ^* E$, is defined as the set of all points $x\in \mathbb {R}^n$ such that $|D\chi _E|(B(x,r))>0$ for all $r>0$ and the limit

6 $$\begin{aligned} \nu _E(x):=-\lim _{r\rightarrow 0}\frac{D\chi _E(B(x,r))}{|D\chi _E|(B(x,r))} \end{aligned}$$

exists in $\mathbb {R}^n$ with $|\nu _E(x)|=1$. The vector $\nu _E(x)$ is called the *measure-theoretic unit normal* to *E* at *x*.

The set of points of density *t* of *E* is defined as

$$\begin{aligned} E^{(t)}=\left\{ x\in \mathbb {R}^n\mid \lim _{r\rightarrow 0^+}\frac{|E\cap B(x,r)|}{|B(x,r)|}=t \right\} , \end{aligned}$$

It is clear that the interiors of *E* and $\mathbb {R}^n\setminus E$ satisfy $E^\circ \subset E^{(1)}$ and $(\mathbb {R}^n\setminus E)^\circ \subset E^{(0)}$, respectively. Moreover, Federer’s theorem (see e.g. [37, Theorem 16.21] or [2, Theorem 3.61]) states that $\partial ^* E\subset E^{(1/2)}$. As an example, for the disk with a slit $\{(r,\theta ): 0<r<1,\, 0<\theta <2\pi \}$, the slit $\{(r,0): 0<r<1\}$ is a part of the topological boundary but not contained in the reduced boundary, since any point of the slit has density 1.

Observe that $\Omega$, $O_1$ and $O_2$ are all sets of finite perimeter since they are open sets with smooth boundary (see e.g. [54, Remark 5.4.2]) and their reduced boundaries coincide with the topological boundaries. In fact, we have the following:

### Remark 3.1

Let $E\subset \mathbb {R}^n$ be a bounded open set with smooth (or $C^1$) boundary, and $n_E$ be the classical outward unit normal vector on $\partial E$. Then $\partial ^*E = \partial E$ and $\nu _E=n_E$.

For the convenience of readers, we include a short proof. Let $x\in \partial E$ and $r>0$. Using [54, Remark 5.4.2], for any open subset $\Omega \subset \mathbb {R}^n$, $|D\chi _E|(\Omega ) = {\mathcal {H}}^{n-1}(\Omega \cap \partial E)$. By the classical Gauss-Green theorem (see e.g. [37, Theorem 9.3]) and the definition of $D\chi _E$, for any $\varphi \in C_0^\infty (\mathbb {R}^n)$,

$$\begin{aligned} -\int _{\mathbb {R}^n} \varphi \,\textrm{d}(D\chi _E) = \int _{E} \nabla \varphi = \int _{\partial E}\varphi n_E \,\textrm{d} {\mathcal {H}}^{n-1} = \int _{\partial E}\varphi n_E \,\textrm{d} |D\chi _E|. \end{aligned}$$

Due to Riesz’s theorem (see e.g. [37, Theorem 4.7]), we have $D\chi _E = -n_E |D\chi _E|$, and hence $D\chi _E(B(x,r))=-\int _{B(x,r)\cap \partial E} n_E \,\textrm{d}{\mathcal {H}}^{n-1}$. Since $n_E$ is smooth (or continuous) on $\partial E$, it follows from definition (6) that $\nu _E$ exists and $\nu _E=n_E$ everywhere on $\partial E$.

The next lemma states that the set ${\mathcal {V}}$ defined in Section 2 is a set of finite perimeter.

### Lemma 3.2

The set ${\mathcal {V}}$ defined by (4) is a set of finite perimeter.

### Proof

Since $O_1$ and $O_2$ have smooth boundary and $\partial {\mathcal {V}}\subset \partial O_1\cup \partial O_2$, if holds that

$$\begin{aligned} {\mathcal {H}}^{n-1}(\partial {\mathcal {V}})\le {\mathcal {H}}^{n-1}(\partial O_1\cup \partial O_2)<\infty . \end{aligned}$$

Due to [2, Proposition 3.62], we conclude that ${\mathcal {V}}$ has finite perimeter. $\square$


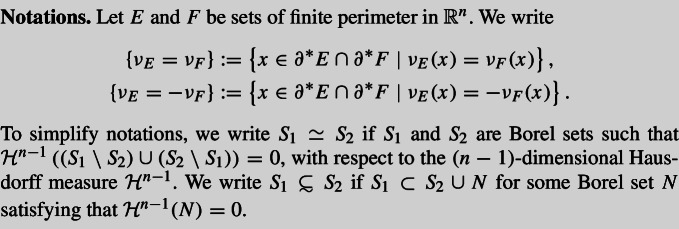


Lemma 2.4 shows that $\partial {\mathcal {V}}\setminus \partial O_2\subset \partial G_0$, it is natural to ask if the reduced boundary of these sets also preserves this relation. We remark that in general, for three Borel sets *A*, *B* and *C*, $\partial A\setminus \partial B\subset \partial C$ does not implies that $\partial ^* A\setminus \partial ^* B\subset \partial ^* C$ since ${\mathcal {H}}^{n-1}(\partial C\setminus \partial ^* C)$ may not be 0. In our setting, the following lemma states that the reduced boundary satisfies this relation up to an ${\mathcal {H}}^{n-1}$-measure zero set:

### Lemma 3.3

, where $G_0,{\mathcal {V}}$ are defined by $(3),(4)$.

### Proof

By [37, Theorem 16.3], we have

$$\begin{aligned} \partial ^*{\mathcal {V}}=\partial ^*({\mathcal {V}}\setminus G_0)\simeq \left( G_0^{(0)}\cap \partial ^* {\mathcal {V}}\right) \cup \left( {\mathcal {V}}^{(0)}\cap \partial ^* G_0\right) \cup \left\{ \nu _{\mathcal {V}}=-\nu _{G_0}\right\} . \end{aligned}$$

It is enough to show that $G_0^{(0)}\cap \partial ^* {\mathcal {V}}\subset \partial O_2=\partial ^* O_2$. To get a contradiction, suppose there exists a point $p\in G_0^{(0)}\cap \partial ^* {\mathcal {V}}$ and $p\not \in \partial O_2$. Since $\partial ^*{\mathcal {V}}\subset \partial {\mathcal {V}}\subset \partial G_0\cup \partial O_2=(\partial G_0\cap \partial O_1)\cup \partial O_2$, there holds $p\in \partial G_0\cap (\partial O_1\setminus \partial O_2)\cap G_0^{(0)}$. Thus, for any $\varepsilon >0$, there exists a small enough neighborhood $U_p^\varepsilon$ of *p*, such that $\frac{|U_p^\varepsilon \cap G_0|}{|U^\varepsilon _p|}\le \varepsilon$. According to Lemma 2.2, we have $U_p^\varepsilon \setminus G_0 \subset \overline{O_1}$. Hence $\frac{|U_p^\varepsilon \cap O_1|}{|U_p^\varepsilon |}=\frac{|U_p^\varepsilon \cap \overline{O_1}|}{|U_p^\varepsilon |}\ge 1-\varepsilon$. However, this contradicts with the fact that $p\in \partial ^* O_1\subset O_1^{(1/2)}$ due to Federer’s theorem. $\square$

Finally we show that the measure-theoretic unit normal on $\partial ^*{\mathcal {V}}$ coincides almost everywhere with the unit normal defined on $\partial ^* O_1$ or $\partial ^* O_2$, but with the opposite direction in the latter case. This is an application of the structure theory for sets of finite perimeter.

### Proposition 3.4

$$\quad \partial ^* {\mathcal {V}}\simeq \left\{ \nu _{{\mathcal {V}}}=\nu _{O_1}\right\} \cup \left\{ \nu _{{\mathcal {V}}}=-\nu _{O_2}\right\} .$$

### Proof

According to Lemma A2 in Appendix A, we have $\partial ^*{\mathcal {V}}\cap \partial ^* G_0\simeq \left\{ \nu _{\mathcal {V}}=-\nu _{G_0} \right\}$ and $\partial ^*{\mathcal {V}}\cap \partial ^* O_2\simeq \left\{ \nu _{\mathcal {V}}=-\nu _{O_2} \right\}$ since ${\mathcal {V}}\cap G_0=\emptyset$ and ${\mathcal {V}}\cap O_2=\emptyset$. Moreover, $G_0\cap O_1=\emptyset$ and $G_0\cap O_2=\emptyset$ imply that $\partial ^*G_0\cap \partial ^* O_1\simeq \left\{ \nu _{G_0}=-\nu _{O_1} \right\}$ and $\partial ^*G_0\cap \partial ^* O_2\simeq \left\{ \nu _{G_0}=-\nu _{O_2} \right\}$. By Lemma 3.3, we have $\partial ^*{\mathcal {V}}\subset \partial ^* O_1\cup \partial ^* O_2$ and , then

$$\begin{aligned} \partial ^* {\mathcal {V}}&\simeq (\partial ^*{\mathcal {V}}\cap \partial ^*G_0\cap \partial ^* O_1)\cup (\partial ^*{\mathcal {V}}\cap \partial ^* O_2)\\&=\left( (\partial ^* {\mathcal {V}}\cap \partial ^* G_0)\cap (\partial ^* G_0\cap \partial ^* O_1)\right) \cup (\partial ^*{\mathcal {V}}\cap \partial ^* O_2)\\&\simeq \left( \left\{ \nu _{\mathcal {V}}=-\nu _{G_0} \right\} \cap \left\{ \nu _{G_0}=-\nu _{O_1} \right\} \right) \cup \left\{ \nu _{\mathcal {V}}=-\nu _{O_2} \right\} \\&= \left\{ \nu _{\mathcal {V}}=\nu _{O_1} \right\} \cup \left\{ \nu _{\mathcal {V}}=-\nu _{O_2} \right\} . \end{aligned}$$

$$\square $$

## Inverse Obstacle Problem for the Laplace Equation

In this section, we prove Theorem 1. Assume $O_1\ne O_2$, say, $O_1\not \subset O_2$ without loss of generality. Let $G_0,{\mathcal {V}}$ be the sets defined by (3),(4) at the beginning of Section 2.

### Lemma 4.1

$$u_1=u_2$$ in $$\overline{G_0}$$.

### Proof

Since $u_1,u_2$ and their normal derivatives coincide on an open subset $\Gamma \subset \partial \Omega$, by the unique continuation for elliptic equation (and path-connectedness), $u_1=u_2$ in $G_0$. They also coincide on $\partial G_0$ due to the regularity $u_1,u_2\in C^1(\overline{G_0})$. $\square$

### Proof of Theorem 1

We notice $u_2$ satisfies $\Delta u_2=0$ on ${\mathcal {V}}$ as ${\mathcal {V}}\cap \overline{O_2}=\emptyset$. On $\partial {\mathcal {V}} \cap \partial O_2$, the boundary condition applies: $u_2\partial _{\nu _2} u_2=0$, where $\nu _2$ is the unit normal vector of $\partial O_2$. As $\partial {\mathcal {V}} \setminus (\partial {\mathcal {V}}\cap \partial O_2)\subset \partial O_1$ by Lemma 2.4, we know $u_1\partial _{\nu _1} u_1=0$ on $\partial {\mathcal {V}}\setminus \partial O_2$, where $\nu _1$ is the normal vector of $\partial O_1$. Since $u_2\in C^1({\overline{\Omega }}\setminus O_2)\cap H^2(\Omega \setminus \overline{O_2})$ is defined on $\Omega \setminus \overline{O_2}$ and extends to a neighborhood of $\Omega \setminus \overline{O_2}$ in the same regularity class (by the standard Sobolev extension), we can apply the Gauss-Green formula in [11, Proposition 6.4] to $u_2$ in the set of finite perimeter ${\mathcal {V}}$,

7 $$\begin{aligned} \int _{{\mathcal {V}}} u_2 \Delta u_2 + \int _{{\mathcal {V}}} |\nabla u_2|^2= \int _{\partial ^* {\mathcal {V}}} u_2 \nabla u_2 \cdot {\nu } \,\textrm{d}{\mathcal {H}}^{n-1}. \end{aligned}$$

Here $\partial ^* {\mathcal {V}}$ is the reduced boundary of ${\mathcal {V}}$, where the measure-theoretical unit normal $\nu$ to ${\mathcal {V}}$ is defined.

Using Proposition 3.4 and Signorini condition at $\partial O_2$, we have

8 $$\begin{aligned} u_2 \nabla u_2 \cdot {\nu }=-u_2\partial _{\nu _2} u_2=0 \quad \text { a.e. on }\,\partial ^* {\mathcal {V}} \cap \partial O_2. \end{aligned}$$

If $\partial ^* {\mathcal {V}}\setminus \partial O_2=\emptyset$, we have

$$\begin{aligned} u_2 \nabla u_2 \cdot {\nu }=-u_2\partial _{\nu _2} u_2=0 \quad \text { a.e. on }\,\partial ^* {\mathcal {V}}. \end{aligned}$$

For the complement, suppose $\partial ^* {\mathcal {V}}\setminus \partial O_2\ne \emptyset$, then take any point $z\in \partial ^*{\mathcal {V}}\setminus \partial O_2$. According to Lemma 2.4, we have $z\in \partial ^*{\mathcal {V}}\setminus \partial O_2\subset \partial {\mathcal {V}}\setminus \partial O_2\subset \partial G_0\cap (\partial O_1\setminus \partial O_2)$. Thus, by Lemma 2.2, for small enough neighborhood $U_z$ of *z*, there holds $U_z^-:=U_z\setminus \overline{O_1}\subset G_0$.

In $U_z^-\subset G_0\subset \Omega \setminus (\overline{O_1\cup O_2})$, both $u_1$ and $u_2$ are defined and of $C^1$. Since $u_1=u_2$ in $U_z^-$ by Lemma 4.1, we have $u_1=u_2$ and $\partial _{\nu _1} u_1=\partial _{\nu _1}u_2$ at *z*. As the choice of *z* is arbitrary, we have

$$u_1=u_2,\text { and }\partial _{\nu _1} u_1=\partial _{\nu _1}u_2 \quad \text { on } \partial ^*{\mathcal {V}}\setminus (\partial ^* {\mathcal {V}} \cap \partial O_2).$$

Then by Proposition 3.4 and Signorini boundary condition for $u_1$ on $\partial O_1$, we have

9 $$\begin{aligned} u_2 \nabla u_2 \cdot \nu =u_2 \partial _{\nu _1} u_2 =u_1 \partial _{\nu _1} u_1= 0 \quad \text { a.e. on }\,\partial ^*{\mathcal {V}}\setminus (\partial ^* {\mathcal {V}} \cap \partial O_2). \end{aligned}$$

Combining (8) and (9), and the case $\partial ^*{\mathcal {V}}\setminus \partial O_2=\emptyset$, we have shown that

$$\begin{aligned} u_2 \nabla u_2 \cdot \nu =0 \quad \text { a.e. on }\,\partial ^*{\mathcal {V}}. \end{aligned}$$

Thus, from (7) and $\Delta u_2=0$ in ${\mathcal {V}}$, it follows that $\int _{{\mathcal {V}}}|\nabla u_2|^2=0$, and consequently $u_2$ is a constant in ${\mathcal {V}}$. This implies that the boundary value *f* is a constant on $\partial \Omega$ by Lemma 2.4 and unique continuation, which contradicts that *f* is not constant on $\partial \Omega$ and thus conclude the proof of Theorem 1. $\square$

## Inverse Obstacle Problem for Elasticity System

In this section, we extend the result we obtained in Section 4 to the Láme system. Let ${\boldsymbol{u}}_1$ and ${\boldsymbol{u}}_2$ be the solution of (2) with obstacles $O_1$ and $O_2$ respectively. Provided that $\boldsymbol{\sigma }({\boldsymbol{u}}_1) {\boldsymbol{n}}|_\Gamma =\boldsymbol{\sigma }({\boldsymbol{u}}_2) {\boldsymbol{n}}|_\Gamma$ for an open subset $\Gamma$ of $\partial \Omega$, we see that ${\boldsymbol{u}}_1$ and ${\boldsymbol{u}}_2$ coincide in $\overline{G_0}$, analogous to Lemma 4.1.

### Lemma 5.1

$${\boldsymbol{u}}_1={\boldsymbol{u}}_2$$ in $$\overline{G_0}$$.

### Proof

Since ${\boldsymbol{u}}_1|_\Gamma ={\boldsymbol{u}}_2|_\Gamma$ and $\boldsymbol{\sigma }({\boldsymbol{u}}_1) {\boldsymbol{n}}|_\Gamma =\boldsymbol{\sigma }({\boldsymbol{u}}_2) {\boldsymbol{n}}|_\Gamma$, by the unique continuation for local Cauchy data [19, Corollary 2.2] and the path-connectedness of $G_0$, there holds ${\boldsymbol{u}}_1={\boldsymbol{u}}_2$ in $G_0$. Moreover, ${\boldsymbol{u}}_1$ and ${\boldsymbol{u}}_2$ also coincide on $\partial G_0$ due to the regularity ${\boldsymbol{u}}_1,{\boldsymbol{u}}_2\in C^1(\overline{G_0})$. $\square$

Our next objective is to prepare Green’s formula for the stress tensor on ${\mathcal {V}}$. To this end, we generalize Green’s formula [48, eq. (4.22)] from Lipschitz domains to sets of finite perimeter based on [11].

### Lemma 5.2

Let *E* be an open set in $\mathbb {R}^n$ and ${\boldsymbol{u}}\in \left( C^1(E)\right) ^n\cap \left( H^2(E)\right) ^n$. Let $U\subset \subset E$ be an open set of finite perimeter. Then

10 $$\begin{aligned} \int _U \boldsymbol{\sigma }({\boldsymbol{u}}): \boldsymbol{\varepsilon }({\boldsymbol{u}})\ \textrm{d}x+\int _U {\boldsymbol{u}}\cdot \text {div }\boldsymbol{\sigma }({\boldsymbol{u}})\ \textrm{d}x=\int _{\partial ^* U}\boldsymbol{\sigma }({\boldsymbol{u}})\boldsymbol{\nu }\cdot {\boldsymbol{u}}\ \textrm{d}{\mathcal {H}}^{n-1}, \end{aligned}$$

where $\boldsymbol{\nu }$ is the measure-theoretic unit normal on the reduced boundary $\partial ^* U$ of *U*.

### Proof

Since $\boldsymbol{\varepsilon }({\boldsymbol{u}})=\frac{1}{2}\left( \nabla {\boldsymbol{u}}+(\nabla {\boldsymbol{u}})^T\right)$, then to prove (10), it is sufficient to show that

11 $$\begin{aligned} {\left\{ \begin{array}{ll} \int _U \boldsymbol{\sigma }({\boldsymbol{u}}): \nabla {\boldsymbol{u}}\ \textrm{d}x+\int _U {\boldsymbol{u}}\cdot \text {div }\boldsymbol{\sigma }({\boldsymbol{u}})\ \textrm{d}x=\int _{\partial ^* U}\boldsymbol{\sigma }({\boldsymbol{u}})\boldsymbol{\nu }\cdot {\boldsymbol{u}}\ \textrm{d}{\mathcal {H}}^{n-1} \\ \\ \int _U \boldsymbol{\sigma }({\boldsymbol{u}}): (\nabla {\boldsymbol{u}})^T\ \textrm{d}x+\int _U {\boldsymbol{u}}\cdot \text {div }\boldsymbol{\sigma }({\boldsymbol{u}})\ \textrm{d}x=\int _{\partial ^* U}\boldsymbol{\sigma }({\boldsymbol{u}})\boldsymbol{\nu }\cdot {\boldsymbol{u}}\ \textrm{d}{\mathcal {H}}^{n-1} \end{array}\right. } \end{aligned}$$

As $\boldsymbol{\sigma }({\boldsymbol{u}})$ is symmetric, the above two equations are exactly the same. Write $\boldsymbol{\sigma }_i=(\sigma _{i1},\sigma _{i2},\cdots ,\sigma _{in})$ the *i*-th row of the matrix $\boldsymbol{\sigma }({\boldsymbol{u}})$, then proving (11) amounts to showing that

12 $$\begin{aligned} \int _U \boldsymbol{\sigma }_i\cdot \nabla u_i\ \textrm{d}x+\int _U u_i\ \text {div }\boldsymbol{\sigma }_i \ \textrm{d}x=\int _U \text {div }(u_i\boldsymbol{\sigma }_i)\ \textrm{d}x=\int _{\partial ^* U}(\boldsymbol{\sigma }_i\cdot \boldsymbol{\nu }) u_i \ \textrm{d}{\mathcal {H}}^{n-1},\ 1\le i\le n.\nonumber \\ \end{aligned}$$

Recall that

13 $$\begin{aligned} \sigma _{ij}=\mu \left( \frac{\partial u_i}{\partial x_j}+\frac{\partial u_j}{\partial x_i}\right) +\delta _i^j \lambda \left( \sum _{k=1}^{n}\frac{\partial u_k}{\partial x_k}\right) , \end{aligned}$$

where $\mu ,\lambda \in C^\infty (E)$. Since ${\boldsymbol{u}}\in (H^2(E))^n$, (13) implies that $\text {div }\boldsymbol{\sigma }_i\in L^2(E)$, in particular, one can view $|\text {div }\boldsymbol{\sigma }_i|$ as a real Radon measure on $\Omega$ and thus $\boldsymbol{\sigma }_i$ is a divergence measure field [11, Definition 2.3]. Notice that $\boldsymbol{\sigma }_i\in C^0(E;\mathbb {R}^n)$ and $u_i\in C^1(E)$, then by [11, Proposition 6.3], there holds (12) and thus we obtain (10). $\square$

### Proof of Theorem 2

Without loss of generality, we assume $O_1\not \subset O_2$. We denote the measure-theoretical unit normal of ${\mathcal {V}}$, $O_1$ and $O_2$ by $\boldsymbol{\nu }$, $\boldsymbol{\nu }_1$, and $\boldsymbol{\nu }_2$, respectively.

Using the Signorini boundary condition on $\partial O_2$ and Proposition 3.4, we have

14 $$\begin{aligned} \boldsymbol{\sigma }({\boldsymbol{u}}_2)_\tau =0,\ ({\boldsymbol{u}}_2)_{\nu }\boldsymbol{\sigma }({\boldsymbol{u}}_2)_{\nu }=({\boldsymbol{u}}_2)_{\nu _2}\boldsymbol{\sigma }({\boldsymbol{u}}_2)_{\nu _2}=0 \quad \text {a.e. on }\ \partial ^*{\mathcal {V}}\cap \partial O_2. \end{aligned}$$

Analogous to the argument used in the proof for Theorem 1 in Section 4, we can obtain

$$\begin{aligned} \boldsymbol{\sigma }({\boldsymbol{u}}_2)_\tau =\boldsymbol{\sigma }({\boldsymbol{u}}_1)_\tau =0,\ \boldsymbol{\sigma }({\boldsymbol{u}}_1)_{\nu _1}=\boldsymbol{\sigma }({\boldsymbol{u}}_2)_{\nu _1}\quad \text {on }\partial ^*{\mathcal {V}}\setminus \partial O_2. \end{aligned}$$

By Lemma 2.4, $\partial ^* {\mathcal {V}}\setminus \partial O_2\subset \partial G_0$, and then Lemma 5 reads that ${\boldsymbol{u}}_1={\boldsymbol{u}}_2$ on $\partial ^* {\mathcal {V}}\setminus \partial O_2$. According to Proposition 3.4 and the Signorini boundary condition holds for ${\boldsymbol{u}}_1$ on $\partial O_1$, there holds

15 $$\begin{aligned} \boldsymbol{\sigma }({\boldsymbol{u}}_2)_{\tau }=\boldsymbol{\sigma }({\boldsymbol{u}}_2)_{\tau _1}=&\boldsymbol{\sigma }({\boldsymbol{u}}_1)_{\tau _1}=0, \nonumber \\ ({\boldsymbol{u}}_2)_\nu \boldsymbol{\sigma }({\boldsymbol{u}}_2)_\nu =({\boldsymbol{u}}_2)_{\nu _1}\boldsymbol{\sigma }({\boldsymbol{u}}_2)_{\nu _1}=&({\boldsymbol{u}}_1)_{\nu _1}\boldsymbol{\sigma }({\boldsymbol{u}}_1)_{\nu _1}=0\quad \text {a.e. on }\partial ^*{\mathcal {V}}\setminus \partial O_2. \end{aligned}$$

Combining (14) and (15), we have

$$\begin{aligned} \boldsymbol{\sigma }({\boldsymbol{u}}_2)_{\tau }=0,\ \boldsymbol{\sigma }({\boldsymbol{u}}_2)\boldsymbol{\nu }\cdot {\boldsymbol{u}}_2=({\boldsymbol{u}}_2)_\nu \boldsymbol{\sigma }({\boldsymbol{u}}_2)_\nu =0\quad \text {on }\partial ^*{\mathcal {V}}. \end{aligned}$$

Thanks to the regularity results [33, Theorem 2.2] and [46, Theorem 3.10], there holds ${\boldsymbol{u}}_2\in (C^1({\overline{\Omega }}\setminus O_2))^n\cap (H^2(\Omega \setminus \overline{O_2}))^n$. By the standard Sobolev extension, we can extend ${\boldsymbol{u}}_2$ to a neighborhood *U* of $\Omega \setminus O_2$ such that ${\mathcal {V}}\subset \subset U$ and ${\boldsymbol{u}}_2\in (C^1(U))^n\cap (H^2(U))^n$. Then applying Lemma 5.2, there holds

$$\begin{aligned} \int _{\mathcal {V}}\boldsymbol{\sigma }({\boldsymbol{u}}_2): \boldsymbol{\varepsilon }({\boldsymbol{u}}_2)\ \textrm{d}x&=\int _{\partial ^* {\mathcal {V}}}\boldsymbol{\sigma }({\boldsymbol{u}}_2)\boldsymbol{\nu }\cdot {\boldsymbol{u}}_2\ \textrm{d}{\mathcal {H}}^{n-1} \\&=\int _{\partial ^*{\mathcal {V}}} (\boldsymbol{\sigma }({\boldsymbol{u}}_2)_\tau +\boldsymbol{\sigma }({\boldsymbol{u}}_2)_\nu \boldsymbol{\nu })\cdot {\boldsymbol{u}}_2\ \textrm{d}{\mathcal {H}}^{n-1} \\&=\int _{\partial ^* {\mathcal {V}}}\boldsymbol{\sigma }({\boldsymbol{u}}_2)_\nu ({\boldsymbol{u}}_2)_\nu \ \textrm{d}{\mathcal {H}}^{n-1}=0. \end{aligned}$$

Since

$$\begin{aligned} \boldsymbol{\sigma }({\boldsymbol{u}}_2): \boldsymbol{\varepsilon }({\boldsymbol{u}}_2)=2\mu \boldsymbol{\varepsilon }({\boldsymbol{u}}_2): \boldsymbol{\varepsilon }({\boldsymbol{u}}_2)+\lambda (\text {tr}\boldsymbol{\varepsilon }({\boldsymbol{u}}_2))^2, \end{aligned}$$

where $\mu ,\lambda >0$ in ${\overline{\Omega }}$. We can conclude that $\boldsymbol{\varepsilon }({\boldsymbol{u}}_2)= 0$ in ${\mathcal {V}}$.

According to Lemma A3 in Appendix A, we have ${\boldsymbol{u}}_2={\boldsymbol{c}}+A{\boldsymbol{x}}$ in ${\mathcal {V}}$, where ${\boldsymbol{c}}\in \mathbb {R}^n$ and $A\in \mathbb {R}^{n\times n}$ is constant a skew-symmetric matrix. Then due to the unique continuation for the static elasticity system (see e.g. [4] or [52, Theorem 2.3]), ${\boldsymbol{u}}_2= {\boldsymbol{c}}+A{\boldsymbol{x}}$ in $\Omega \setminus \overline{O_2}$ and ${\boldsymbol{f}}=({\boldsymbol{c}}+A{\boldsymbol{x}})|_{\partial \Omega }$, which contradicts with the assumption ${\boldsymbol{f}}\not \in {\mathcal {R}}$. $\square$

## Counterexamples to Unique Solvability

In this section, we discuss counterexamples to the unique solvability of the inverse Signorini obstacle problem. For the scalar problem, it is not possible to solve the problem if the Dirichlet boundary value *f* is a nonnegative constant. Indeed, in this case the constant function $u=c\ge 0$ is the solution to the Signorini problem by the uniqueness of solution, since $u=c\ge 0$ is allowed by the Signorini condition $u|_{\partial O} \ge 0$. This means that given any nonnegative constant boundary value, the normal derivative of the solution for any Signorini obstacle is identically zero.

As a special case in the setting of Theorem 1, we show that the inverse obstacle problem is uniquely solvable if *f* is a negative constant.

### Proposition 6.1

Let $\Omega \subset \mathbb {R}^n$ be a bounded open set with smooth boundary and $\Gamma \subset \partial \Omega$ be a nonempty open subset. Let $O_1,O_2\subset \subset \Omega$ be open subsets with smooth boundary. Assume that $\Omega \setminus \overline{O_1},\Omega \setminus \overline{O_2}$ are connected. Suppose $u_1,u_2$ solves (1) for the given boundary value *f* with obstacle $O_1,O_2$, respectively. If *f* is a negative constant and $\partial _{n} u_1|_{\Gamma }=\partial _{n} u_2|_{\Gamma }$ with respect to the unit normal ${\boldsymbol{n}}$ to $\partial \Omega$, then $O_1=O_2$.

### Proof

Assume that $O_1\ne O_2$, say that $O_1\not \subset O_2$ without loss of generality. Following the proof of Theorem 1, we have $u_2=c$ for some constant *c* in ${\overline{\Omega }}\setminus O_2$. Under the assumption that *f* is a negative constant, this implies that $u_2=c<0$. However, this contradicts the Signorini condition $u_2|_{\partial O_2}\ge 0$, as long as $O_2\ne \emptyset$. In the case of $O_2=\emptyset$, Lemma 4.1 shows $u_1=u_2=c<0$ on ${\overline{\Omega }} \setminus O_1$, which again contradicts with the Signorini condition $u_1|_{\partial O_1}\ge 0$ as long as $O_1\ne \emptyset$. Hence both $O_1,O_2$ have to be empty set. $\square$

For the elasticity case, the solvability issue is more complicated because the shape of the obstacle matters to the Signorini condition ${\boldsymbol{u}}\cdot \nu \le 0$. Consider the sets

$$\begin{aligned} \Xi := \big \{O\subset \Omega \mid O\text { is a nonempty open subset with smooth boundary} \big \}, \end{aligned}$$

and we define that

$$\begin{aligned} \Upsilon _{A,{\boldsymbol{c}}}:=\big \{O\subset \Xi \mid ({\boldsymbol{c}}+A{\boldsymbol{x}})\cdot \nu _O({\boldsymbol{x}})=0\text { for all }{\boldsymbol{x}}\in \partial O \big \} \cup \{\emptyset \}, \end{aligned}$$

where $\nu _O$ denotes the inward normal of *O* at $\partial O$. Complementing Theorem 2, in the following lemma we characterize the unique solvability in the case of ${\boldsymbol{f}}\in {\mathcal {R}}$.

### Proposition 6.2

Let $\Omega \subset \mathbb {R}^n$ be a bounded open set with smooth boundary and $\Gamma \subset \partial \Omega$ be a nonempty open subset. Let $O_1,O_2\subset \subset \Omega$ be open subsets with smooth boundary. Assume that $\Omega \setminus \overline{O_1},\Omega \setminus \overline{O_2}$ are connected. Suppose ${\boldsymbol{u}}_1, {\boldsymbol{u}}_2$ solves the elasticity system (2) for ${\boldsymbol{f}}={\boldsymbol{c}}+A{\boldsymbol{x}}\in {\mathcal {R}}$ with $O_1, O_2$, respectively, for some vector ${\boldsymbol{c}}\in \mathbb {R}^n$ and skew-symmetric matrix *A*. If $\boldsymbol{\sigma }({\boldsymbol{u}}_1){\boldsymbol{n}}|_{\Gamma }=\boldsymbol{\sigma }({\boldsymbol{u}}_2){\boldsymbol{n}}|_{\Gamma }$, then $O_1=O_2$ unless $O_1,O_2\in \Upsilon _{A,{\boldsymbol{c}}}$.

### Proof

It suffices to show that $O_1\ne O_2$ implies $O_1,O_2\in \Upsilon _{A,{\boldsymbol{c}}}$. Assume $O_1\not \subset O_2$ without loss of generality. Following the proof of Theorem 2 and the continuity of solutions, we have ${\boldsymbol{u}}_2={\boldsymbol{c}}+A{\boldsymbol{x}}$ in ${\overline{\Omega }}\setminus O_2$.

Consider the case when $O_2\ne \emptyset$. The function ${\boldsymbol{u}}_2$ clearly extends smoothly to ${\widetilde{{\boldsymbol{u}}}}_2:={\boldsymbol{c}}+A{\boldsymbol{x}}$ in the whole domain ${\overline{\Omega }}$. Then we apply the divergence theorem to ${\widetilde{{\boldsymbol{u}}}}_2$ in $O_2$,

$$\begin{aligned} \int _{\partial O_2}({\boldsymbol{c}}+A{\boldsymbol{x}})\cdot \nu _{O_2}({\boldsymbol{x}}) =-\int _{O_2}\text {div }({\boldsymbol{c}}+A{\boldsymbol{x}})=0. \end{aligned}$$

Since the Signorini condition for ${\boldsymbol{u}}_2$ on $\partial O_2$ requires that

$$\begin{aligned} ({\boldsymbol{u}}_2)_\nu =({\boldsymbol{c}}+A{\boldsymbol{x}})\cdot \nu _{O_2}({\boldsymbol{x}})\le 0, \quad \text {for }{\boldsymbol{x}}\in \partial O_2, \end{aligned}$$

we have $({\boldsymbol{c}}+A{\boldsymbol{x}})\cdot \nu _{O_2}({\boldsymbol{x}})=0$ on $\partial O_2$ and thus $O_2\in \Upsilon _{A,{\boldsymbol{c}}}$. As ${\boldsymbol{u}}_2={\boldsymbol{c}}+A{\boldsymbol{x}}$ in ${\overline{\Omega }}\setminus O_2$, it follows that $\boldsymbol{\sigma }({\boldsymbol{u}}_2)=0$ identically and thus $\boldsymbol{\sigma }({\boldsymbol{u}}_1){\boldsymbol{n}}|_{\Gamma }=\boldsymbol{\sigma }({\boldsymbol{u}}_2){\boldsymbol{n}}|_{\Gamma }=0$. Then the unique continuation property yields that ${\boldsymbol{u}}_1={\boldsymbol{c}}+A{\boldsymbol{x}}$ in ${\overline{\Omega }}\setminus O_1$. Then the same argument as above gives $O_1\in \Upsilon _{A,{\boldsymbol{c}}}$ (since $O_1\ne \emptyset$ as $O_1\not \subset O_2$). In the case of $O_2=\emptyset$, one repeats the argument for ${\boldsymbol{u}}_1$ to show $O_1\in \Upsilon _{A,{\boldsymbol{c}}}$. $\square$

A corollary of Proposition 6.2 is that if $\Upsilon _{A,{\boldsymbol{c}}}= \{\emptyset \}$, then the inverse obstacle problem is uniquely solvable with boundary data ${\boldsymbol{f}}={\boldsymbol{c}}+A{\boldsymbol{x}}$. The vector ${\boldsymbol{c}}$ and matrix *A* determine if $\Upsilon _{A,{\boldsymbol{c}}}$ is empty or not. Next, we give examples for both cases $\Upsilon _{A,{\boldsymbol{c}}}\ne \{\emptyset \}$ and $\Upsilon _{A,{\boldsymbol{c}}}= \{\emptyset \}$.

### Example 6.3

For any given constant skew-symmetric matrix *A* and a point $p\in \Omega$, we set ${\boldsymbol{c}}=-Ap$. Then there exists a ball *B* centered at *p* with radius $\delta >0$ such that $B\subset \Omega$. Notice that for any $q\in \partial B(p,\delta )$, the normal derivative $\nu _B$ at *q* is $(q-p)/\delta$. Since *A* is skew-symmetric, we have

$$\begin{aligned} ({\boldsymbol{c}}+Aq)\cdot \nu _B(q)=\frac{1}{\delta }A(q-p)\cdot (q-p)=0. \end{aligned}$$

Therefore, $$B(p,\delta )\in \Upsilon _{A,{\boldsymbol{c}}}$$.

### Example 6.4

For any given constant skew-symmetric matrix *A*, the range of *A* restricted in $\Omega$ is bounded, i.e. for a large enough *M*, we have

$$\begin{aligned} \max _{{\boldsymbol{x}}\in \Omega }\, |A{\boldsymbol{x}}|<M. \end{aligned}$$

Then we choose a vector ${\boldsymbol{c}}$ with $|{\boldsymbol{c}}|>M$. Assume that there exists a nonempty set $O\in \Upsilon _{A,{\boldsymbol{c}}}$. To get a contradiction, we choose a point $z\in \partial O$ such that $\nu _O(z)={\boldsymbol{c}}/|{\boldsymbol{c}}|$. Such a point *z* can be chosen in the following way. Let *P* be a hyperplane perpendicular to ${\boldsymbol{c}}$. Initially place the hyperplane *P* disjoint from the set *O*, and move the hyperplane towards *P* until they first intersect. At any intersection points, it must satisfy that *P* is tangential to $\partial O$ so that the normal directions of *P* and $\partial O$ coincide. Then at point *z*, we have

$$\begin{aligned} |({\boldsymbol{c}}+A z)\cdot \nu _O(z)|\ge |{\boldsymbol{c}}|-|Az\cdot \frac{{\boldsymbol{c}}}{|{\boldsymbol{c}}|}|> |{\boldsymbol{c}}|-M>0. \end{aligned}$$

However, this contradicts with the assumption that $({\boldsymbol{c}}+A {\boldsymbol{x}})\cdot \nu _O({\boldsymbol{x}})=0$ for all ${\boldsymbol{x}}\in \partial O$. Thus $\Upsilon _{A,{\boldsymbol{c}}}= \{\emptyset \}$.

## Data Availability

Data sharing is not applicable to this article as no datasets were generated or analyzed during the study.

## Auxiliary Lemmas

### Lemma A1

Let $A, B\subset \mathbb {R}^n$ be two nonempty connected open sets satisfying $A\subset B$. If $\partial A\subset \partial B$, then $A=B$.

### Proof

Assume $A\ne B$, then there exists a point $p\in B$ but $p\not \in A$. If $p\in {\overline{A}}$, then $p\in ({\overline{A}}\setminus A)=\partial A\subset \partial B$ and $p\not \in B$, a contradiction. On the contrary, if $p\not \in {\overline{A}}$, then $p\in B\setminus {\overline{A}}\ne \emptyset$, and we have

$$\begin{aligned} (B\setminus {\overline{A}})\cup A=B\setminus \partial A\supset B\setminus \partial B=B. \end{aligned}$$

Notice that $(B\setminus {\overline{A}})\cap A=\emptyset$. Thus, the set *B* can be written as the union of two disjoint open sets $B\setminus {\overline{A}}$ and *A*. As $A\ne \emptyset$, it must satisfy that $A=B$ due to the connectedness of *B*. $\square$

### Lemma A2

If *E* and *F* are sets of finite perimeter, then

$$\begin{aligned} \partial ^* E\cap \partial ^* F\simeq \left\{ \nu _E=\nu _F \right\} \cup \left\{ \nu _E=-\nu _F \right\} . \end{aligned}$$

Furthermore, if $E\cap F=\emptyset$, then

$$\begin{aligned} \partial ^* E\cap \partial ^* F\simeq \left\{ \nu _E=-\nu _F \right\} . \end{aligned}$$

### Proof

According to [37, Theorem 16.3], we have

$$\begin{aligned} \partial ^*(E\cap F)&\simeq \left( F^{(1)}\cap \partial ^* E\right) \cup \left( E^{(1)}\cap \partial ^* F\right) \cup \left\{ \nu _E=\nu _F\right\} ,\\ \partial ^*(E\setminus F)&\simeq \left( F^{(0)}\cap \partial ^* E\right) \cup \left( E^{(1)}\cap \partial ^* F\right) \cup \left\{ \nu _E=-\nu _F\right\} ,\\ \partial ^*(E\cup F)&\simeq \left( F^{(0)}\cap \partial ^* E\right) \cup \left( E^{(0)}\cap \partial ^* F\right) \cup \left\{ \nu _E=\nu _F\right\} . \end{aligned}$$

Since $E\cap F$, $E\cup F$ and $E\setminus F$ are all sets of finite perimeter (see e.g. [37, Lemma 12.22]), we can obtain

$$\begin{aligned} \partial ^* E&=\partial ^*\left( (E\setminus F)\cup (E\cap F)\right) \\&\simeq \left( (E\setminus F)^{(0)}\cap \partial ^*(E\cap F)\right) \cup \left( (E\cap F)^{(0)}\cap \partial ^*(E\setminus F)\right) \cup \left\{ \nu _{E\setminus F}=\nu _{E\cap F}\right\} \\&\subset \partial ^*(E\cap F)\cup \partial ^* (E\setminus F) \end{aligned}$$

as $\left\{ \nu _{E\setminus F}=\nu _{E\cap F}\right\} \subset \partial ^*(E\cap F)\cap \partial ^* (E\setminus F)$ by definition.

By Federer’s theorem, $\partial ^* E\cap \partial ^* F\subset E^{(1/2)}\cap F^{(1/2)}$. Therefore we have

Since $\left\{ \nu _E=\nu _F\right\} \cup \left\{ \nu _E=-\nu _F\right\} \subset \partial ^* E\cap \partial ^* F$ by definition, then there follows $\partial ^* E\cap \partial ^* F\simeq \left\{ \nu _E=\nu _F \right\} \cup \left\{ \nu _E=-\nu _F \right\}$.

Similarly, when $E\cap F=\emptyset$, i.e., $E=E\setminus F$, we have $\partial ^* E=\partial ^* (E\setminus F)$ and $\partial ^* E\cap \partial ^* F\simeq \left\{ \nu _E=-\nu _F \right\}$ follows. $\square$

### Lemma A3

For any open subset $U\subset \subset \Omega$, if ${\boldsymbol{u}}\in (H^2(U))^n\cap (C^1(U))^n$ satisfies $\boldsymbol{\varepsilon }({\boldsymbol{u}})=0$, then ${\boldsymbol{u}}={\boldsymbol{c}}+A{\boldsymbol{x}}$ in *U*, where ${\boldsymbol{c}}\in \mathbb {R}^n$ is a constant vector and $A\in \mathbb {R}^{n\times n}$ is a constant skew-symmetric matrix.

### Proof

Writing that ${\boldsymbol{u}}=(u_1,u_2,\cdots ,u_n)$, for $1\le i,j\le n$, $\boldsymbol{\varepsilon }({\boldsymbol{u}})=\nabla {\boldsymbol{u}}+(\nabla {\boldsymbol{u}})^T=0$, we get that

$$\begin{aligned} \frac{\partial u_i}{\partial x_i}=0,\ \frac{\partial u_j}{\partial x_i}=-\frac{\partial u_i}{\partial x_j}. \end{aligned}$$

Since $u_k\in H^2(U)$ for $1\le k\le n$, it holds that

$$\begin{aligned} \frac{\partial ^2 u_k}{\partial x_i\partial x_j}=\frac{\partial ^2 u_k}{\partial x_j\partial x_i} \;\text { in } L^2(U). \end{aligned}$$

Therefore, for any $1\le i,j,k\le n$, we have

$$\begin{aligned} \frac{\partial ^2 u_k}{\partial x_i\partial x_j}=- \frac{\partial ^2 u_i}{\partial x_k\partial x_j}= \frac{\partial ^2 u_j}{\partial x_i\partial x_k}=- \frac{\partial ^2 u_k}{\partial x_i\partial x_j}=0. \end{aligned}$$

This shows that $\frac{\partial u_i}{\partial x_j}$ is constant for all *i*, *j*, and thus $\nabla {\boldsymbol{u}}\in \mathbb {R}^{n\times n}$ is a constant skew-symmetric matrix. Due to ${\boldsymbol{u}}\in (C^1(U))^n$, we obtain ${\boldsymbol{u}}={\boldsymbol{c}}+A{\boldsymbol{x}}$ in *U* with a constant vector ${\boldsymbol{c}}\in \mathbb {R}^n$ and a constant skew-symmetric matrix $A=\nabla {\boldsymbol{u}}\in \mathbb {R}^{n\times n}$. $\square$
